# Visual Phenotype Matching: Cues to Paternity Are Present in Rhesus Macaque Faces

**DOI:** 10.1371/journal.pone.0055846

**Published:** 2013-02-22

**Authors:** Anahita J. N. Kazem, Anja Widdig

**Affiliations:** 1 Junior Research Group of Primate Kin Selection, Department of Primatology, Max-Planck Institute for Evolutionary Anthropology, Leipzig, Germany; 2 Department of Biology, Norwegian University of Science & Technology, Trondheim, Norway; 3 Institute of Biology, Behavioural Ecology Research Group, University of Leipzig, Leipzig, Germany; Université de Strasbourg, France

## Abstract

The ability to recognize kin and thus behaviourally discriminate between conspecifics based on genetic relatedness is of importance both in acquiring inclusive fitness benefits and to enable optimal inbreeding. In primates, mechanisms allowing recognition of paternal relatives are of particular interest, given that in these mating systems patrilineal information is unlikely to be available via social familiarity. Humans use visual phenotype matching based on facial features to identify their own and other's close relatives, and recent studies suggest similar abilities may be present in other species. However it is unclear to what extent familial resemblances remain detectable against the background levels of relatedness typically found within demes in the wild – a necessary condition if facial cues are to function in kin recognition under natural conditions. Here, we experimentally investigate whether parent-offspring relationships are discernible in rhesus macaque (*Macaca mulatta*) faces drawn from a large free-ranging population more representative of the latter scenario, and in which genetic relatedness has been well quantified from pedigrees determined via molecular markers. We used the human visual system as a means of integrating multiple types of facial cue simultaneously, and demonstrate that paternal, as well as maternal, resemblance to both sons and daughters can be detected even by human observers. Experts performed better than participants who lacked previous experience working with nonhuman primates. However the finding that even naïve individuals succeeded at the task underlines the strength of the phenotypic cues present in faces.

## Introduction

The ability to recognize kin and thus behaviourally discriminate between conspecifics based on cues that correlate with genetic relatedness is taxonomically widespread, being documented from bacteria to humans (reviewed in[1]–[3]). Kin recognition can facilitate the acquisition of inclusive fitness benefits [4] through nepotism, the preferential treatment of close relatives in prosocial interactions and the investment of care [5]. It also functions in the context of mate choice [6], [7] to enable optimal inbreeding, in which the inclusive fitness benefits of mating with close kin are balanced against the costs of inbreeding depression, via selection of mates of intermediate relatedness [8], [9].

In many primates, including humans, a long period of postnatal association between mothers and their offspring means that a contextual cue such as prior association (“familiarity”) between individuals is a reasonable guide to identify close maternal relatives. However, the fact that females often mate with multiple partners during their likely conception period leads to paternity uncertainty, making the Shakespearean adage that “it is a wise father that knows his own child” as applicable in other primates as it is to humans. As expected, humans report and provide more assistance to kin than nonkin, are more inclined to help close over distant relatives, and tend to avoid close consanguineous matings [10], [11]. In Western societies the investment strategies amongst networks of more distant kin additionally exhibit a matrilateral bias, perhaps because the genetic links between any pair of relatives through male lineages are more uncertain than those through females ([12], [13]; see also [14], [15] for an alternative explanation).

Many macaque and baboon species reside in large mixed-sex groups characterized by female philopatry and male dispersal, and promiscuous mating by both sexes. Here too there is ample evidence of favouritism toward maternal kin; rates of association, grooming and coalition support are higher for close maternal relatives than other categories of partner [16], [17]. Importantly, there is evidence that these nepotistic biases can translate into fitness benefits. In chacma baboons, females who form stronger social bonds with their mothers, daughters and maternal half-sisters survive longer and have higher offspring survivorship than do less socially integrated females within the same troop [18], [19].

Nevertheless, in wild and free-ranging populations of cercopithecines evidence is also accumulating for behavioural discrimination of paternal kin. For example, adult female rhesus macaques and savannah baboons direct more affiliation toward their paternal half-sisters than to unrelated females [20]–[22], juvenile mandrills affiliate more with adults who are paternally related (fathers and paternal half-sisters) than others [23], and male savannah baboons selectively support their own offspring in agonistic disputes [24]. This latter example appears to be an instance of true paternal care. Furthermore, presence of the genetic father in a group is associated with accelerated maturation of his offspring, an important component of lifetime reproductive success [25]. Finally, there are indications from a range of species of reduced probabilities of mating between close paternal relatives if these individuals co-reside after reaching sexual maturity ([26]–[28]; but see [29]).

The mechanisms by which paternal kin are recognized in primates remain puzzling (reviewed in [30]). One possibility is phenotype matching, in which individuals identify their relatives by matching conspecifics' phenotypic characteristics to a template derived from either the individual's own phenotype or that of known kin [31], [32]. This allows recognition of previously unfamiliar relatives and, as the cues used correlate with the genetic similarity between individuals, can produce a graded response toward kin of differing degrees of relatedness. A variety of phenotypic traits may be used. For example olfactory cues are a commonly used mechanism in many rodents [33], and their importance is now gaining recognition in primates [34], [35]. In humans, visual phenotype matching has been reported. Many facial features are heritable, and close relatives often resemble one another (for recent quantitative genetic studies see [36]–[38] and references therein). In experimental games people exhibit greater trust and cooperation toward computer-generated images that resemble themselves, as well as judging the sexual attractiveness of opposite-sex self-resembling images to be lower [39]. Humans can also detect kin resemblances between two unfamiliar individuals based on their faces [40], [41], with the perceived degree of similarity depending on the level of relatedness between the individuals pictured [42]. That such assessments may have fitness consequences is demonstrated by studies in natural populations, showing that fathers invest more in children perceived to resemble them more closely [43], and that the frequency of spousal and child abuse by men is inversely related to how often others have told them that their children resembled them [44].

These findings have generated interest in whether other primates share similar abilities. Monkey faces, as in many other taxa [45], are individually distinctive and discriminated by the animals themselves [46], [47]. Craniofacial measurements are also known to be heritable, for example in rhesus macaques [48]. Visual phenotype matching could allow direct recognition between relatives who encounter each other for the first time, or might supplement information obtained via other modalities (for example comparing the face of an unknown individual to that of a group member already identified as one's relative via postnatal association or self-similarity in odour). This would be beneficial in a number of contexts, for example allowing females to identify and avoid mating with immigrant males to which they are paternally related, or enabling emigrating males to distinguish and associate with relatives (fathers, maternal or paternal half-brothers) that already reside in their new group. Identifying physical resemblances between third parties can be useful even when both are unrelated to oneself, allowing immigrants to predict the kin and likely alliance partners of unfamiliar individuals before incurring the costs of interacting with the individuals concerned. This seems plausible as macaques and baboons can distinguish affiliations between third parties in both photographs [49] and vocalizations [50], and are known to use such information in managing their social conflicts, for example selectively recruiting allies that are unrelated to their current opponent [51], or accepting a “reconciliatory” vocalization from a close relative of a recent opponent as a proxy for direct reconciliation by the opponent itself [52].

There is evidence that humans can detect mother-offspring kinship in the faces of several primate species, predominantly apes [53], [54]. Only one study has gone further [55], experimentally examining the ability to detect paternal relatedness in a small sample of chimpanzees and rhesus macaques – a potentially more interesting scenario given that under natural conditions patrilineal information is less likely to be available via social familiarity [30]. Following extensive training in an onscreen match-to-sample task, both species succeeded in discriminating adult mother-offspring and father-offspring dyads from unrelated individuals. For facial cues to function in kin recognition under natural conditions, however, familial resemblances must be detectable even against the background levels of relatedness found within demes in the wild. Features such as migration between neighbouring groups and patterns of group fission along family lineages produce genetic structuring in populations. Therefore closely related dyads may have to be discriminated from “nonkin” individuals some of whom are distantly related to (and somewhat facially similar to) the dyads in question. Existing studies have predominantly used stimuli from captive populations, and in some cases many of the nonkin referents were drawn from other colonies, meaning the coefficient of relatedness (*r*) between a parent and the nonkin “decoy” individual is likely to be effectively zero. This could exaggerate the disparity between the kin and nonkin dyads used. Moreover, individuals held at the same institution experience common environmental conditions (e.g. diet, social networks, degree of outdoor exposure), which could enhance the facial similarity of these dyads, relative to the nonkin referents taken from elsewhere (cf. “phenotypic convergence”, [42], [56]). Interestingly, the only species in which a negative result has so far been obtained used images from a wild baboon population with appreciable background relatedness [54].

Here, we investigate whether resemblance between offspring and parents of both sexes can be detected in the faces of rhesus macaques from a large free-ranging population with background relatedness more representative of natural populations, and in which genetic relatedness has been drawn from an extensive pedigree determined via molecular markers. Given the current lack of knowledge about whether it is variation in shape of the face or specific features, coloration of the skin, pelage or eyes, or texture and subtle markings on the face that might be most informative concerning relatedness, we used the human visual system as a simple means of integrating multiple cues simultaneously in facial images. If even heterospecifics succeed, this provides a strong test of whether the cues required for visual phenotype matching are present in macaque faces.

## Methods

### Stimuli

Digital colour photographs of rhesus macaques were collected under free-ranging conditions at Cayo Santiago (18°09' N, 65°44' W), Puerto Rico, an island colony of approximately 850 individuals residing in 6 naturally-formed social groups [57]. Facial images were taken in two orientations, frontal (both face and gaze directed at the camera) and three-quarter view (left side of the face oriented approximately 45° away from the camera and gazing straight ahead of the face), when the animal was sitting and with a neutral expression. We obtained the majority of images at a distance of 1.5–3.0 m from the animal under even lighting conditions (open shade). All animals in this population are identifiable via unique identification tattoos and ear notch combinations.

Image backgrounds were masked using Adobe Photoshop (CS4 v. 11.0.2), by drawing a continuous line around the head encompassing features such as cheek whiskers, jowls and crests, and then magnifying the image and feathering this outline in order to capture the detailed edges of the fur. Although ear shape and colour potentially convey information about relatedness the ears were masked at the hairline, as animals in this population have identification notches which are assigned randomly with regard to the relatedness between animals but might nevertheless influence similarity ratings. The masking procedure was performed blind with respect to the eventual triad membership or kinship category of the image. All faces were then centred and standardized to a head height of 400 pixels, occupying a vertical distance of 10.5 cm when displayed onscreen, and placed on a black background.

### Experimental design

**Figure 1:**
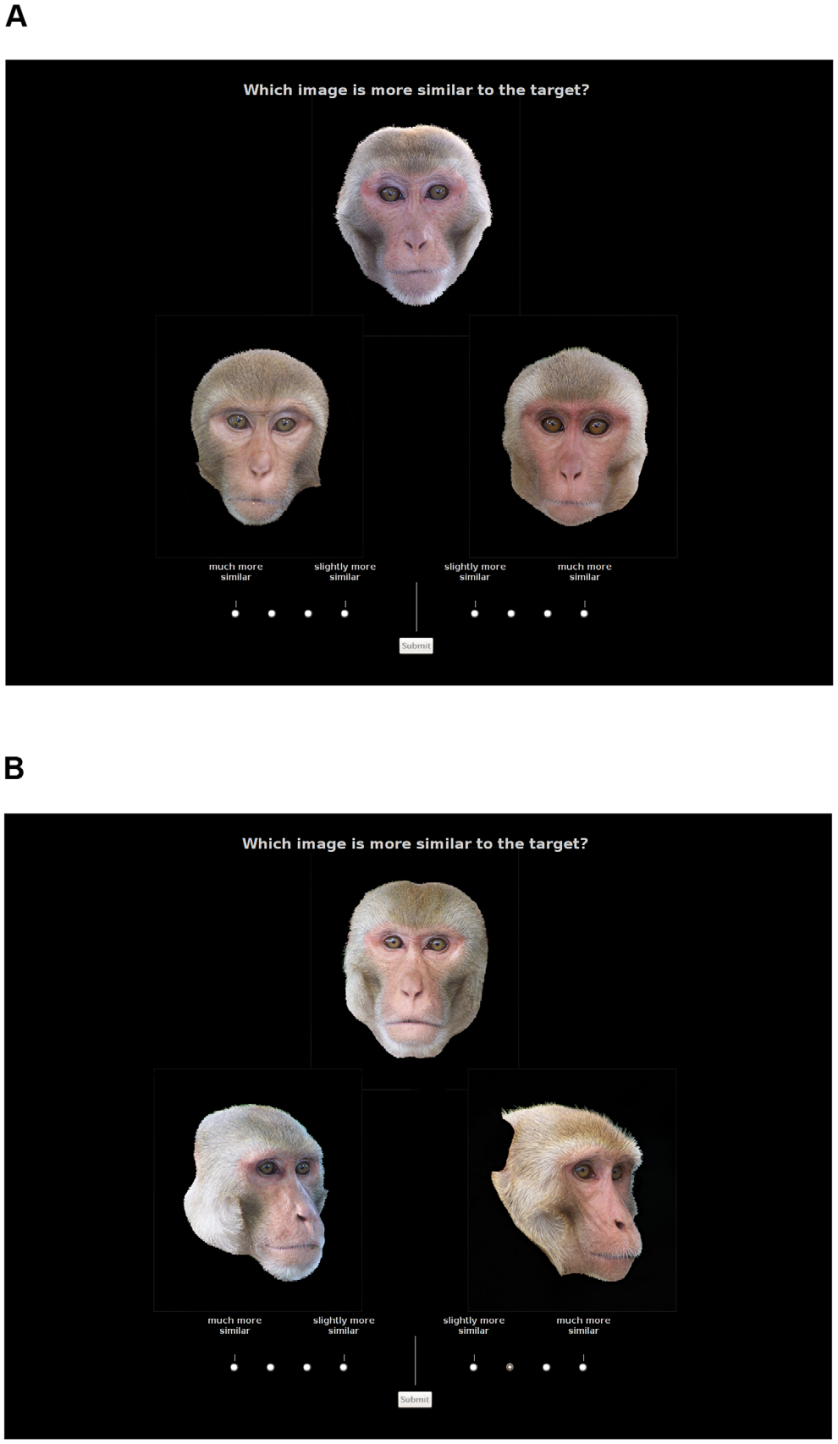
Examples of (A) kin discrimination and (B) individual discrimination trials. The target is displayed uppermost, with two alternative matches placed below. (A) A paternal line mixed-sex kin discrimination trial, with the father displayed uppermost. In this case the correct choice (daughter) is the lower-right image. (B) A male-male individual discrimination trial, in which the target and lower-left images are of the same animal.

A within-subjects design was utilized in which two factors, parental line (maternal, paternal) and offspring sex, were combined to produce four types of kin discrimination trial (mother-daughter, mother-son, father-daughter, father-son). Human raters (hereafter subjects) were asked to identify which of two macaque faces viewed on a computer monitor was more similar to a target face displayed simultaneously at the top of the screen. In kin discrimination (KD) image triads, the target animal was always a parent and one of the potential matches was its offspring. The other was an unrelated “decoy” individual (see below for relatedness definitions), of the same sex and age as the offspring (≤ 2 years age difference), with the two images also being matched for general lighting conditions. All individuals depicted were sexually mature (≥4 years old), and the distribution of ages represented amongst parents and offspring was similar across the four treatment conditions. All faces were shown in frontal orientation (Fig. 1A). A total of 32 trials were presented, eight in each of the four conditions. Due to the limited number of genetically assigned sires who possessed a high quality image, five male images were re-used (once as the target in a father-daughter trial and once as target in a father-son trial, in each case); target image ID was therefore incorporated as a random factor in analyses. All remaining stimuli were used only once (hence a total of 91 different animals were used).

To check that participants could reliably distinguish different macaques under the experimental conditions, a series of 12 individual discrimination (ID) trials were also presented. Here the target was displayed in frontal orientation, and the correct match was a photograph of the same individual with its face turned at 45° away from the camera (¾ orientation, Fig. 1B). The non-match was a different (and unrelated) individual of the same sex and age (≤2 years difference), also pictured in ¾-view, and with the images matched for general lighting conditions. Equal numbers of male versus female trials were presented, and within each sex in half the trials the pair of ¾-view images were facing right and in half facing left. All individuals pictured were sexually mature, and the age distribution of triads was chosen so as to encompass the full range of ages of the parents and offspring used in the KD trials.

Subjects indicated their decision using an 8-point response scale at the bottom of the screen, in which either the left or right match was selected as being “more similar to the target” (see Fig. 1). A forced-choice paradigm was used as studies on human kin recognition have suggested that subjects are more likely to select the correct image when forced to guess the most likely match from a set of possibilities, than if they are asked to assign a similarity rating to each of the alternative stimuli [58]. We used a combination of these methods, which allows for a more subtle weighted analysis than does a simple binary response. Our response scale ranged outwards from the centre with each of the two possible match images represented by 4 points, anchored at “slightly more similar” and “much more similar” (than was the alternative image) to the target. Thus, when selecting one of the images as the best match, subjects also indicated the relative degree of perceived resemblance between each of the two alternatives and the target.

In both KD and ID trials, the target was always positioned in the upper centre of screen and the position of the correct match was randomized left-right. Each participant received all 48 trials, in a unique order, with a restricted randomization procedure being used to ensure that the four types of KD trial and four types of ID trial were interleaved evenly throughout the session. The task was controlled via a dedicated web-based presentation system (written in Java and Perl), linked to a MySQL database.

### Human raters and procedure

The experiment was conducted between September – November 2010. Fifty-nine participants (30 female, 29 male) were recruited, ranging from 19 to 59 years of age (mean  = 30, SD  = 7.65 years). Of these, 35 (59.3%) participants had worked with nonhuman primates in the past whilst 24 had not (“expert” versus “inexperienced” groups, respectively). None were familiar with the macaque population used as stimuli, and all were unaware of the hypothesis being tested.

Subjects completed the task at individual computer terminals. A brief questionnaire was completed first, including the subject's age and gender, previous experience working with nonhuman primates (categorized as either none, or ≤3, ≤6, ≤12, ≤24 or >24 months in duration), the taxa involved (at species level; later coded as simians, anthropoids or both), and presence/absence of any visual impairment. Visual acuity of all participants was normal or corrected-to-normal. One practice trial followed, involving a “mock” individual discrimination trial using the faces of three different males, two of them somewhat facially similar to each other. The main task was then divided into two halves, separated by an interval of 1–2 minutes. Participants were allowed as much time as desired to make decisions. The dataset has been deposited in the Dryad repository: http://dx.doi.org/10.5061/dryad.q6r01.

### Genetic sampling and genotyping

Genetic data are taken from a database for this population first implemented in 1992 and continuously extended [20], [59]–[61]. The database currently consists of 2302 animals genotyped on average at 14.62±2.44 loci (± SD) out of a total of 21 STR markers. Using CERVUS 3.0 [62], we found the mean number of alleles per locus was 7.38±2.87, mean polymorphic information content was 0.69±0.80, mean expected heterozygosity was 0.74±0.07 and mean observed heterozygosity across loci was 0.75±0.08. There was no evidence of a null allele occurring at these loci and all except one locus (D20S206) were in Hardy-Weinberg equilibrium (HWE). The deviation from the HWE at this locus could be due to chance, mutation or typing errors. The overall typing error rate derived from (at least one) mother-offspring mismatch was 11% in the entire database, however that of the subset used in this study was only 2.2%. DNA samples used in this study were exclusively blood samples extracted using the DNEasy Blood & Tissue kit (Qiagen Inc., Valencia, CA, USA).

### Assigning parentage

Maternity (from behavioural observations), date of birth and sex of individuals are known from the long-term demographic database of the Caribbean Primate Research Center (CPRC). A total of 91 individuals were pictured in our kin discrimination trials. Dams were determined genetically for all but four of these individuals (95.6%), for whom no maternal sample was available. Genetic samples were also available for a total of 58.8% of their grand-dams, and all of these confirmed the prior behavioural data. Given that in our entire database only 1.8% of behaviourally-assigned mothers were not subsequently confirmed genetically (potentially due to sample swaps, or misidentification of the behavioural mother due to adoption or kidnapping, A. Widdig, unpublished data), we felt confident in accepting the behaviourally-determined mother in those cases where samples were not available for a mother or grandmother.

For paternity assignment, all males who had been of reproductive age [63] and present on the island at least 200 days before a given infant's birth [64] were considered as potential sires for a given infant. Males in the entire population fulfilling these criteria were included in the paternity analyses in order to account for extra-group paternities observed in this population [65]. Our analysis included only those cases in which a given mother-father-offspring trio were genotyped on at least 12 common loci or, if lacking a sample or genotypes of mothers were restricted, father-offspring duos had to be genotyped on at least 15 common loci. We used a combination of exclusion and likelihood analyses. Overall, we solved paternity for 85 (93.4%) of the 91 individuals pictured in this study. For 79 of these the assigned sire had no mismatch with the respective mother-offspring pair, and all other candidate sires could be excluded on at least two loci. In the remaining six cases the assigned sire had no mismatch with the respective mother-offspring pair, but one other candidate sire could only be excluded at one locus. For the latter cases, all paternity assignments were additionally supported at the 95% confidence level by the maximum likelihood method calculated by CERVUS 3.0. The six individuals with unresolved paternity (all were targets, i.e. the parental individual in the triad, in our experiment) were born before systematic sampling began in 1992, increasing the chance that not all their potential sires had been sampled. The potential sires we were able to test were all excluded by multiple mismatches, suggesting that the actual fathers were not sampled.

We also aimed to assign the maternal and paternal grandfathers for each of our 91 animals. For the 86 genetically known dams, we were able to determine 55 maternal grandfathers (63.9%). In 51 of these cases we could exclude all candidate sires but one with 2 mismatches, and in the remaining 4 cases we could exclude all candidate sires but one with 1 mismatch. This corresponds to 60.4% of known maternal grandfathers for all 91 stimulus individuals. Likewise, based on the 85 cases of solved paternity we determined a total of 41 paternal grandfathers (48.2%). In 38 of these cases we could exclude all candidate sires but one with 2 mismatches, and in the remaining 3 cases we could exclude all candidate sires but one with 1 mismatch. This corresponds to 45.1% of known paternal grandfathers for our 91 individuals. Again, all paternity assignments based on exclusion at one locus were additionally supported at the 95% confidence level by the maximum likelihood method. Note that, despite some missing paternity information in the pedigree, we were still able to estimate the relatedness between the individuals selected as triads (see below).

### Calculating kinship for triad selection

We then used a pedigree-based approach, based on the above parentage assignments, to establish parent-offspring dyads. When selecting triads, the “target” individual was definitively assigned as a parent of the individual chosen as the “offspring” image (*r*≥0.5). However additions to the wider pedigree after this study was conducted revealed an updated estimate of *r* = 0.313 for one dyad that had originally been classified as sire-daughter; given that the two individuals were nevertheless related through the paternal line and their *r*-value was substantially greater than between the target and decoy individuals (nonkin, *r* < 0.001), the triad was retained in the analysis. To be selected as the “unrelated decoy” image in a given trial, our aim was that the individual was unrelated for a minimum of two generations to both the target and offspring individuals, i.e. possess no parents or grandparents in common with either animal (*r*<0.063). This was achieved in all except one case (where the target-decoy shared one ancestor, hence *r* = 0.063).

In cases where the sire or grandsire of an individual was unknown (see above), we used an exclusion rule to ensure that a given dyad was indeed unrelated. We first identified all potential sires for a given subject based on the reproductive males present at the time of conception (using census records), reduced by all males actually excluded as the sire using genotypic data. This provided a list of potential sires that could not be excluded (e.g. due to a lack of samples or power). The identities of the non-excluded potential sires of a given subject A were then compared with the identities of the assigned sire and grandsires of subject B, and if no overlap occurred we considered this dyad to be unrelated. If an overlap occurred, an expected relatedness was calculated based on the probability of sharing one or several ancestors and accounting for the empirical level of inbreeding avoidance in this population (Mundry & Kulik, unpublished data). In all cases, the expected *r*-value obtained was <0.063, therefore meeting our criterion for being “unrelated”.

### Ethics statement

The Cayo Santiago macaques comprise a free-ranging colony on an uninhabited island. The animals subsist on a combination of natural vegetation, supplemented once daily with commercially available monkey chow provided at a number of locations. Water is available *ad libitum* via both rainfall and drinking fountains distributed throughout the site. Handling is limited to an annual 2-month trapping period conducted by the site management, during which new yearlings are provided with identification codes and physiological samples may be collected for research purposes from specific individuals, which are then released. During this period a number of juvenile animals are also permanently removed and housed at other facilities, in order to counterbalance the high birthrates and a lack of predators. Blood samples were obtained by temporarily immobilizing individuals using intramuscular injections of 10 mg/ kg body weight of Hydrochloride Ketamine and two 2 ml blood samples then drawn via femoral venipuncture (a single 2 ml sample in the case of infants). Collection of images and genetic samples was approved by the CPRC and the Institutional Animal Care and Use Committee (IACUC) of the University of Puerto Rico (protocol No. 4060105). Human participation in the computer task was entirely voluntary, written consent was obtained and the study was conducted in accordance with the Declaration of Helsinki.

### Statistical analysis

Responses on the 8-point scale were assigned values from −3.5 through to +3.5 (in increments of 1.0), where a positive sign indicates selection of the correct match image in a trial. We first checked whether each participant could identify individual macaques at above chance levels, by conducting one sample t-tests separately for each person to assess whether their response scores on the 12 individual discrimination trials differed from zero. All exhibited positive mean scores, and 56 of 59 participants (94.9%) succeeded (three tests failed to reach significance; all other *p*-values ≤0.033). However, there was no significant correlation between participants' mean response scores in the individual discrimination and kin discrimination trials (Pearson correlation, r = 0.04, N = 59, *p* = 0.791). Inspection of the data also revealed that there was no consistent difference between the mean kin discrimination scores of these particular three subjects and the others. As performance in the two types of task was not correlated, all participants were retained for the subsequent kin discrimination analyses.

Previous experience working with nonhuman primates was entered as a binary variable in all models (see below), as there was no correlation between the participants' duration of previous experience (in months, six categories) and either mean individual discrimination (Spearman correlation: r_s_ = 0.19, N = 59, *p* = 0.154) or mean kin discrimination response scores (r_s_ = 0.18, N = 59, *p* = 0.183). However an unpaired t-test revealed that, in the case of kin discrimination, participants who possessed previous experience had higher response scores than those without (mean ± SD: experts  = 0.76±0.331, inexperienced  = 0.57±0.270; t(57)  = 2.25, *p*<0.029).

Generalized Linear Mixed Models [66] with Gaussian error and identity link were used to investigate whether response scores in the kin discrimination task differed significantly from zero (i.e. chance performance – no consistent preference for either the related or unrelated match). Note that the way in which response scores were coded leads to the *intercept* being the critical term in the model; a positive and significant intercept demonstrates that participants prefer the related (offspring) over the unrelated (decoy) image. Line (paternal versus maternal kin line), triad type (same- or mixed-sex) and previous experience (yes/no) were included as the main fixed effects, together with the key interaction term of line*triad type. The three-way interaction for line*triad type*experience (plus its remaining lower-order constituent terms) was also included. We checked for any influence of participant gender, trial position in the sequence (in case longitudinal effects of increasing familiarization with the task, or alternatively fatigue effects, were present), and the position in which the correct match was presented onscreen in a given trial (left or right), by including these “control” variables as fixed effects, together with the interaction term for trial position*experience. Values for trial position (covariate) were z-transformed beforehand to a mean of 0 and standard deviation of 1. GLMMs for the individual discrimination task followed a similar format, but triad sex (all-male vs all-female) was the main fixed effect, and hence the only interaction terms were triad sex*experience and trial position*experience.

Participant identity, image triad identity, and the identity of the images used as the target, left match and right match in each case, were always included as random effects. To control for the possibility that subjects might differ in how their performance changes over the course of the experiment (trial position), and that such subject-specific changes might depend on an individual's overall performance, the initial kin discrimination GLMM included the terms for random slopes of performance against trial position within subjects and for an intercept-slope correlation across subjects [67]. The fit of this model did not differ significantly from a reduced model without the correlation term (likelihood ratio test, χ^2^ = 1.61, d.f.  = 1, *p* = 0.204), which was therefore removed before testing for an effect of the random slopes component, which also proved nonsignificant (χ^2^ = 0.67, d.f. = 1, *p* = 0.412). Our full model therefore uses a random effects structure that accounts only for random intercepts.

Our approach entailed first establishing the overall significance of the full kin discrimination model [68], as compared with a null model (here, one that excludes the main predictors of interest and participant experience and their interactions). Having demonstrated significance of the full model, we checked for significant predictors by first dropping all non-significant interaction terms, and then the non-significant main effects (factors showing nonsignificant trends were retained). This produces a final model that retains all the fixed effect(s) of primary interest, together with any main effects or interactions that were significant. At each stage, the fit of the current model was compared with that of the reduced version using a likelihood ratio test. As the main goal is to test whether the overall intercept is significantly positive, the model was then re-run with all nonsignificant fixed effects excluded (if a factor had a significant effect, this test was performed separately for each of the factor's levels).

GLMM analyses were performed using R v. 2.9.2 [69], using the function “lmer” provided by the library “lme4” [70], and all other analyses using IBM SPSS v. 19.0. All tests were two-tailed with the alpha level set at *p* = 0.05. To achieve a more reliable *p*-value in GLMMs, models were fitted using Maximum Likelihood rather than Restricted Maximum Likelihood [71]. In GLMMs, *p*-values were calculated using Markov Chain Monte Carlo (MCMC) simulations [66] using the function “pvals.fnc” from the R package “languageR” [72]. If a pMCMC value obtained was very close to 0.05, its accuracy was improved by increasing the number of simulations used from 10,000 to 100,000.

## Results

### Individual discrimination

**Figure 2:**
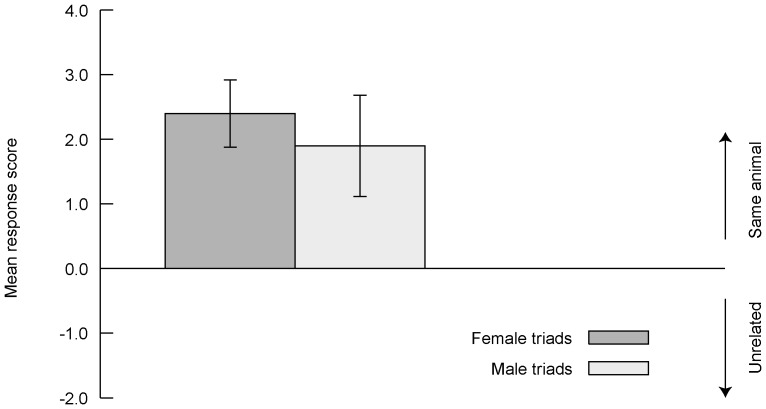
Performance on individual discrimination trials. Mean (and SD) resemblance scores between an alternative view of the same- versus a different (and unrelated) individual and the target image, in male-male and female-female triads. A mean of zero represents chance performance. Positive values indicate that a second image of the same individual was rated as being more similar to the target animal than was an equivalently-oriented image of an unrelated individual, with higher values indicating a greater disparity in perceived similarity.

The GLMM examining performance in distinguishing individual macaque faces demonstrated that there was no significant difference in response scores for male-male versus female-female triads (triad sex coded as female = 0 male = 1, estimate  = −0.66, SE = 0.472, *p* = 0.195), nor any significant effect of the four remaining variables (presence/absence of experience with nonhuman primates, and the “control” variables participant gender, trial position in sequence, and side on which the correct match was presented; all *p*-values ≥0.642). The interaction terms in the full model were not significant (Table 1b). Dropping the interaction terms therefore produced the final model (shown in Table 1a), which confirms the absence of any significant main effects. Excluding these allows the estimate for the intercept in the overall sample to be examined and revealed that the intercept was both positive and highly significant (Table 2, Fig. 2), as might be expected given the pattern of individual t-tests described in the Methods. Finally, subjects' performance was significantly better on individual discrimination (mean ± SD response score  = 2.14±0.539) than kin discrimination trials (mean ± SD  = 0.68±0.319; paired t-test: N = 59, t(58)  = −18.18, *p*<0.001).

**Table 1:** Results of models examining performance in the individual discrimination task.

Variable	Estimate	HPD95	HPD95	Pmcmc
		lower	upper	
a)				
Intercept	2.302	1.589	3.051	
Triad sex (female = 0, male = 1)	−0.542	−1.528	0.391	0.242
Experience (no = 0, yes = 1)	0.185	−0.084	0.447	0.181
Participant gender (female = 0, male = 1)	−0.001	−0.275	0.251	0.982
Trial position	−0.025	−0.132	0.087	0.681
Side correct image (left = 0, right = 1)	0.002	−0.253	0.264	0.929
b)				
Triad sex (male): experience (yes)	0.199	−0.252	0.648	0.381
Trial position: experience (yes)	−0.070	−0.285	0.160	0.546

(a) The final model. The initial full model had contained the variables listed in (a) together with (b) the interaction terms. Parameter estimates are provided from the last model in which a given factor or interaction term remained included.

**Table 2:** Estimates of overall intercept (mean response score) in the individual discrimination (ID) and kin discrimination (KD) tasks.

Task	Subjects	Estimate	HPD95	HPD95	pMCMC
			lower	upper	
ID trials	All	2.141	1.645	2.627	**<0.001**
KD trials	Expert	0.756	0.481	1.033	**<0.001**
	Inexperienced *	0.576	0.211	0.927	**0.002**

Estimates are from final GLMMs in which all significant effects have been controlled and all nonsignificant factors dropped. Kin discrimination models were therefore run separately for expert versus inexperienced participants. *For the inexperienced subjects, the significant factor ‘trial position’ was also included in the final model (estimate  = 0.23, SE  = 0.074, pMCMC <0.002); trial position values were standardized beforehand (to mean  = 0 and SD  = 1) and hence do not affect the estimate of overall intercept. Significantly positive estimates for the intercept indicate performance at greater than chance levels, and are highlighted in boldface.

### Kin discrimination

Before proceeding we confirmed the overall significance of the full model, as compared with a null model that excluded the main predictors of interest, the known effect of experience and their interactions (likelihood ratio test: χ^2^ = 17.60, d.f.  = 8, *p* = 0.025). From the full model, the nonsignificant three-way interaction term (see Table 3d) was dropped, followed by the lower-order interaction terms line*triad type and triad type*experience (neither of which were significant; Table 3c), and finally the nonsignificant “control” variables (participant gender and side on which the correct match was presented; Table 3b). The pattern of results for all other factors and interaction terms was qualitatively similar in each of these model stages to that produced in the final reduced model, which is presented in Table 3a.

**Table 3:** Results of models examining performance in the kin discrimination task**.**

Variable	Estimate	HPD95	HPD95	pMCMC
		lower	Upper	
a)				
Intercept	0.169	−0.365	0.686	
Line (maternal = 0, paternal = 1)	0.471	−0.171	1.087	0.137
Triad type (mixed sex = 0, same sex = 1)	0.340	−0.252	0.902	0.245
Experience (no = 0, yes = 1)	0.334	0.111	0.559	**0.003**
Trial position	0.247	0.117	0.376	**<0.001**
Line (paternal): experience (yes)	−0.304	−0.612	0.004	0.054
Trial position: experience (yes)	−0.192	−0.344	−0.035	**0.015**
b)				
Participant gender (female = 0, male = 1)	−0.073	−0.228	0.093	0.383
Side correct image (left = 0, right = 1)	0.131	−0.034	0.304	0.127
c)				
Line (paternal): triad type (same)	−0.769	−1.871	0.423	0.206
Triad type (same): experience (yes)	0.001	−0.293	0.323	0.986
d)				
Line (paternal): triad type (same): experience (yes)	−0.132	−0.738	0.491	0.699

(a) The final model. (b) – (d) The additional factors and interaction terms that were dropped from earlier successively reduced models. The initial full model is therefore described by the variables (a) – (d). Parameter estimates for a given factor or interaction term are provided from the most recent model in which it remained included. Significant effects are highlighted in boldface.

**Figure 3:**
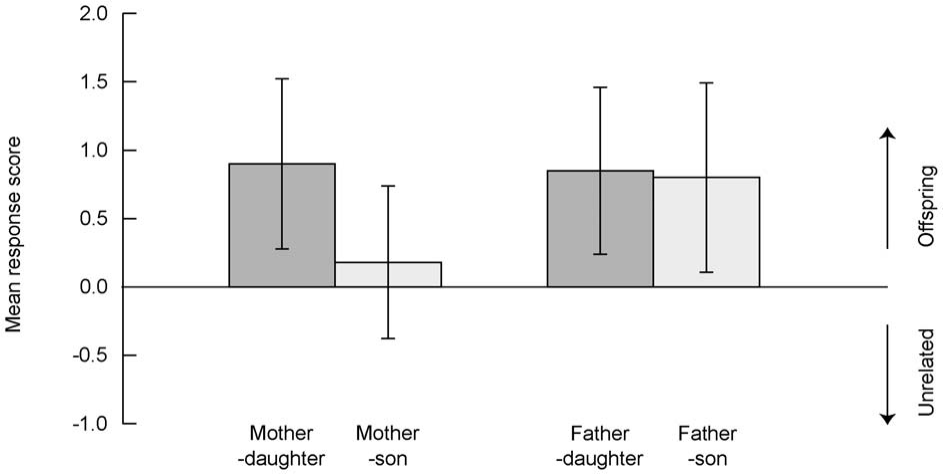
Performance on kin discrimination trials. Mean (and SD) resemblance scores between offspring versus unrelated individuals and the target image (parent), as a function of relatedness type (paternal, maternal) and the subject's level of previous experience with nonhuman primates. A mean of zero represents chance performance. Positive values indicate that the offspring image was rated as being more similar to the parental target than was the unrelated individual, with higher values indicating a greater disparity in perceived similarity.

There was no main effect of relatedness line (paternal versus maternal; Fig. 3), but there was a marginally nonsignificant trend for an interaction between line and the rater's level of previous experience with nonhuman primates (Table 3a), which suggested a tendency for father-offspring trials to be slightly easier than mother-offspring trials, particularly for inexperienced subjects. Performance did not differ significantly between same- or mixed-sex trials in any model (Table 3a; Fig. 3). Finally, there was a significant interaction between trial position and the subject's experience level. Expert participants in general performed at higher levels than those with no previous experience, and their performance did not change significantly over the course of the experiment, whereas inexperienced participants improved strongly as they progressed through the task (Table 3a, also see below).

Given the strong trend for an interaction between relatedness line and the subject's level of previous experience, as well as to check whether both sets of subjects independently succeeded at the kin discrimination task, we explored further by performing GLMMs separately for the expert and inexperienced groups. The only fixed effects included were relatedness line and trial position, as all other factors had been nonsignificant in the main analysis. There was no significant difference in performance between paternal versus maternal trials in either of the models (estimate (maternal = 0, paternal = 1): expert subjects  = 0.17, SE  = 0.280, *p* = 0.540; inexperienced subjects  = 0.47, SE  = 0.385, *p* = 0.196), perhaps owing to the reduction in sample size when testing separately within each of the subject groups. As expected, there was no significant effect of trial position in expert subjects (estimate  = 0.05, SE  = 0.061, *p*  = 0.414), but a strong positive effect in inexperienced subjects (estimate  = 0.23, SE  = 0.074, *p* = 0.002). Dropping the nonsignificant main effect of line allows examination of the estimate for the overall intercept, and demonstrated that the intercept was positive and highly significant in both of the subject groups (Table 2). Therefore both expert and inexperienced participants did on average show a preference for choosing the correct match (i.e. the offspring image).

## Discussion

Participants in this study successfully identified parent-offspring dyads of rhesus macaques on the basis of perceived facial resemblance. Subjects correctly selected the offspring individual's face as being more similar to the parental target than was an age-matched unrelated decoy of the same sex, at levels significantly exceeding chance performance. As the task design ensured that alternative cues such as image background or quality differences were not consistently associated with genetic relationships between the individuals portrayed, the results demonstrate that information regarding parent-offspring relatedness must be present in the face of this species. That even untrained heterospecific observers succeed, and on the basis of the relatively impoverished information available in static two-dimensional images displayed at low resolution, suggests the strength of these phenotypic cues.

Importantly, both father-offspring and mother-offspring pairs were successfully detected. Indeed the presence of a marginally nonsignificant trend for an interaction between participants' prior experience level and the type of parental target suggests that paternal trials may be easier than maternal ones, at least for inexperienced heterospecific observers. This effect requires confirmation, as we cannot exclude the possibility that it was due to the particular examplars available in our stimulus sets, nor that maternally related dyads might contain subtle information which is detectable by a conspecific observer but not by a human. However an overall effect of parental line has been demonstrated in a study which used rhesus macaques as subjects; here too, performance was significantly better on father-offspring than mother-offspring trials [55].

That relatives look alike does not necessarily imply there has been selection to reveal kinship *per se*. Familial resemblance might simply be a byproduct of facial traits being heritable, and selection acting to maintain high phenotypic variation for the purposes of individual discrimination (i.e. kin resemblance is a cue rather than a specialized signal). Conspecifics may nevertheless detect and use those cues to direct their social behaviour. In fact in humans the highly context-specific nature of observers' responses to perceived facial similarity (cooperative responses being higher toward self-resembling faces of both sexes, whilst attractiveness judgements are reduced only toward self-resembling faces of the opposite sex) suggests that the detection of kinship information (if not its production) is more specialized than would be expected if it were a mere byproduct of general face-processing mechanisms [39]. In rhesus, the possibility remains open that facial cues of paternal relatedness have been positively selected, whilst cues of maternity have not or to a lesser degree, perhaps because the latter information is usually already available through other channels such as prior association. This could be achieved via genomic imprinting – the differential expression of paternally- versus maternally-derived alleles – at loci controlling facial phenotype [73], [74]. In species where females mate promiscuously some theoretical models predict that young offspring should not advertise their paternal identity, due to the costs of aggression or infanticidal attacks by adult males that are not the father ([75]; but see [76]). However our images were of sexually mature macaques, an age when individuals are no longer vulnerable to withdrawal of investment or aggression from non-fathers and the potential benefits of detecting paternal kin (see Introduction) likely outweigh such costs.

In Parr and colleagues' study [55] the effect of parental line was accompanied by an effect of offspring sex, performance being higher on trials involving sons than daughters, leading the authors to conclude that in rhesus macaques male faces may simply be more “distinctive” than female faces overall. We did not find this pattern – there was no significant interaction between parental line and triad type (same- versus mixed-sex) in kin discrimination, and participants were as successful discriminating female-female triads as male-male ones in the individual discrimination task. Whilst mindful of the fact that our study used heterospecific subjects, we suggest that caution in interpretation is merited. Whether faces are more variable (hence distinctive) in one sex than the other, whilst in itself an interesting question, would not necessarily generate greater kin resemblances within that sex, as the phenotypic variance may stem from environmental effects rather than a high additive genetic contribution. For example male faces might be more sensitive than females to (non-shared) environmental influences during development, which would produce high individual distinctiveness yet low kin resemblance in this sex. Moreover, when genetic factors do play a large role in facial variability, the sex-specific pattern of dispersal in macaques means that average background relatedness (and hence baseline resemblance) between a randomly selected pair of adults in a social group is likely higher between philopatric females than between males, who are the dispersing sex ([77], reviewed in [78]; likewise, in captive studies males are the sex for which the nonkin referent images are most likely to be obtained from outside colonies). This background relatedness might also be relatively greater via the maternal than the paternal line, although this will depend on additional factors such as the degree of reproductive skew in a population. Thus demographic factors which affect population genetic structure may also provide a (non-adaptive) explanation for lower performance on female-female and/or mother-offspring trials in a match-to-sample task, as greater background resemblance between a female target and the decoy may make it more difficult to pick out the related dyads. Finally, their results might reflect sex-specific perception of kinship cues. Females, the sex expected to suffer higher costs of inbreeding in mammals [79], [80], should be particularly sensitive to relatedness information in *opposite*-sex faces. The majority of rhesus subjects in the Parr et al. study [55] happened to be females, but unfortunately the sample size precluded statistically testing for an effect of subject sex. An interesting question for future studies that use conspecific subjects would be to explore whether kin discrimination performance is affected by an interaction between sex of the observer and that of the individuals pictured.

Little is known about which aspects of the face (e.g. overall configuration or specific features, colour and markings) are most informative regarding genetic similarity. Eye-tracking studies reveal that both macaques and humans show disproportionate fixation on the upper region of conspecific faces, especially the eyes, presumably due to their role in expressing emotions, reciprocating interest and indicating focus of attention [47]. In humans the lower half of the face has been found to have relatively little influence upon kinship judgements about third parties [40], implying that structural similarities between relatives in the upper face and eye regions are of greater importance. Interestingly, a recent study of rhesus macaques has shown that facial measurements are more similar between paternal half-sisters than between age-matched nonkin [81], and some indication that factors describing the upper part of the face exhibited the clearest distinction between the paternal sib group and the control groups. It remains to be seen to what extent this holds for other kin categories.

More extensive morphometric studies would be useful in resolving the above issues. These techniques would allow statistical determination of whether or not paternal line relatives cluster more closely in face space than do maternal relatives of the same degree, whether male kin resemble one another more closely than female kin do, and whether certain facial features (or extracted composite dimensions) correlate better with the coefficient of relatedness than others. Possible interactions, for instance between parental line and facial region, would also be of interest – is there any truth to the proverbial “you have your mother's eyes” (but perhaps your father's nose)? Ultimately, quantitative genetic studies would also be needed to determine whether any differences in the degree of kin resemblance observed between the sexes, or between different facial features, are due to differential heritability and/or differences between sexes or traits in the common environmental contribution to phenotypic variation (relatives share more similar environments than do other individuals). However this requires faciometric data on very large samples of individuals from pedigreed populations, preferably living under natural developmental conditions.

That participants with experience studying nonhuman primates outperformed naïve individuals in kin discrimination is to be expected. Numerous studies of face processing in humans (and other primates) have demonstrated the effect of developmental or adult exposure in sharpening perceptual skills for a given category of faces. Examples include a “conspecific advantage” for discriminating members of one's own species more readily than heterospecifics. Individual monkeys provide a face-like category of stimuli that is no longer discriminated spontaneously by humans beyond the age of 9 months [46], [82], and in explicit recognition tasks adults distinguish unfamiliar human faces more accurately than those of macaques [46], [83]. However, recognition abilities improve if exposure to heterospecifics is accompanied by consistent individuation – infants retain their recognition memory for macaque faces if during exposure each animal is repeatedly referred to by a specific name [84], and trained primate caretakers outperform non-expert adults [83]. Our subjects were significantly better at identifying individual macaques than discerning similarities between pairs of relatives, despite the fact that the individual discrimination task involved the additional processing required to match frontal views with partially rotated faces. The fact that for all but two of the expert participants their prior experience had been in working with great apes suggests that it is the process of differentiating individuals of another species which enhanced kin discrimination, rather than familiarity with macaque faces *per se*. This is also suggested by the finding that the success rates of inexperienced subjects improved markedly over the course of the experimental session, presumably because they acquired increasing familiarity with the subtle variation between macaque faces. It seems that three months of prior experience was sufficient to elevate starting performance in the expert group, as greater durations were not associated with further improvements in performance. This makes sense as in most primatological studies the intensive period of learning animals' identities and dispositions necessarily takes place during the initial phase.

Our results demonstrate that visual cues of both paternal and maternal relatedness exist in rhesus macaques, sufficient to allow discrimination of parent-offspring relationships even in populations with appreciable levels of background relatedness. Both humans (this study) and captive rhesus macaques [55] successfully perform phenotype matching between rhesus faces on the basis of static images. Whether the animals themselves can spontaneously recognize familial facial resemblance under natural conditions, and which features are used, remain to be seen. Furthermore, an ability to recognize finely graded kinship categories can exist without necessarily being reflected in differential treatment of conspecifics [85]. An important question for future studies, therefore, is whether differential behavioural responses occur in response to facial cues or not, and if so, whether these confer functional advantages in nepotistic or mate choice contexts.
